# A Test Case for DNA Barcodes to Identify Species

**DOI:** 10.1371/journal.pbio.0020357

**Published:** 2004-09-28

One hundred years before Darwin returned from his voyage on the *H. M. S. Beagle* “struck with certain facts” that “seemed to throw some light on the origin of species,” Linnaeus published the first systematic taxonomy of life. In *Systema Naturae,* the Swedish botanist divided organisms into plants, animals, and minerals, eventually assigning scientific names to 7,700 plant and 4,400 animal species, and popularizing the binomial system—as in Homo sapiens—of naming species.

In the 1700s and 1800s, naturalists classified organisms based on morphology, devoting their careers to naming newfound plants and animals. Today biologists still use Linnaean taxonomy as the foundation of scientific classification. But with just a fraction of the estimated 5–30 million species on the planet already named and too few specialists to do the job, biologists are looking for high-throughput tools that can rapidly and accurately identify both individuals of a species and entirely new species. That's what some scientists say the DNA barcode will do. The DNA barcode, as the name implies, uses genes to identify species much like supermarket barcodes identify products. The idea is that a short stretch of genetic code from a reference gene is unique enough to one species to distinguish it from every other species, and that comparisons of sequence variations in that stretch of gene can reveal evolutionary relationships among species.

Such technology could radically advance biologists' attempts to achieve the long-standing goal of cataloging life on earth, but the approach is controversial, with critics questioning both the method and its applications. (For more on the debate, see “DNA Barcoding: Promise and Pitfalls,” also in this issue.) Paul Hebert and colleagues offer a proof of the utility of the DNA barcoding concept, using a 648-basepair region of a mitochondrial gene (cytochrome *c* oxidase I, or COI) in a study of 260 North American bird species.

Mitochondria—the cell's power generators—contain their own DNA, and mitochondrial DNA (mtDNA) evolves much faster than nuclear DNA. It evolves so quickly, in fact, that mtDNA sequence variation has been found not just between closely related, or sister, species but also within species. Still, the variation is much greater among than within species, which is why mtDNA divergences have become a tool for identifying species.

Hebert and colleagues tested the effectiveness of the mtDNA COI barcode by matching bird species flagged by the COI barcode against those already established by taxonomic methods. The litmus test for DNA barcoding is absence of COI sequence overlap between species. Beyond that, differences within species should be significantly fewer than those between species. And that's what the researchers found. All 260 species had unique COI barcodes, with differences between species for the most part much more frequent—on average, 18 times more common—than those within species. In the 130 species represented by two or more individuals, COI sequences were either identical or closest to other sequences within that species. For these 260 bird species (of the 667 bird species that breed in North America), the authors report, the COI barcodes “separate individuals into the categories that taxonomists call species.”

The COI barcode, the authors propose, could help resolve problematic classifications based on morphology, as arise when populations of a single species acquire distinct characteristics after geographic barriers prevent their interbreeding. For example, the similar COI sequences found in American and black oystercatchers here support taxonomic studies suggesting that they are actually color morphs of one species. And conversely, highly divergent COI sequences might bolster taxonomic studies indicating that lineages of uncertain status are indeed distinct species.

Future studies will have to determine whether these results can be generalized to animals in other climes and ecosystems, but the authors argue that constructing a comprehensive library of barcodes will facilitate such efforts. Hebert and colleagues conclude that the success of DNA barcoding depends not only on such a repository—with sequences pegged to well-characterized species exemplars—but also on the expertise of trained taxonomists. The hope is that large-scale, standardized testing based on a uniform barcode sequence could go a long way toward finishing what Linnaeus started: a full classification of all plant and animal life. To E. O. Wilson, every species is “a masterpiece of evolution, offering a vast source of useful scientific knowledge because it is so thoroughly adapted to the environment in which it lives.” Faced with what Wilson calls the “worst wave of extinction since the dinosaurs died,” the need for a fast and easy way to identify species has never been greater.

